# Supporting the Delivery of Infection Prevention and Control Training to Healthcare Workers: Insights from the Sector

**DOI:** 10.3390/healthcare10050936

**Published:** 2022-05-18

**Authors:** Mohammed Qureshi, Abrar Chughtai, Holly Seale

**Affiliations:** School of Population Health, University of New South Wales, Sydney 2052, Australia; abrar.chughtai@unsw.edu.au (A.C.); h.seale@unsw.edu.au (H.S.)

**Keywords:** healthcare workers, infection control, infectious disease transmission, occupational health, semi-structured interviews, training programs

## Abstract

Infection prevention and control (IPC) cannot be implemented without healthcare workers (HCWs) being properly trained and competent. The provision of training is essential, yet there is a gap in our understanding of the factors impacting the implementation of IPC training. This paper reports the results from in-depth interviews that explored the current landscape around IPC training delivered across low-, middle-, and high-income countries. Semi-structured interviews were conducted with the key stakeholders involved in policymaking or IPC implementation in Saudi Arabia, Pakistan, India, Indonesia, the Philippines, and Australia. Although the training was mandated for many HCWs, participants indicated that only some training elements were mandatory. Participants spoke about covering various topics, but those in low-resource settings spoke about the challenges of delivering training. Classroom-based training dominated, but online delivery modes were also used in some locations. Whilst HCW’s training was postulated to have improved during the COVID-19 pandemic, the capacity to deliver training did not improve in some settings. More research is needed to establish the essential elements that could underpin the development of training packages.

## 1. Introduction

It has been suggested that, when there is good adherence to infection prevention protocols by frontline healthcare workers (HCW), the risk of transmission to patients and other staff members appears low. However, healthcare settings continue to report outbreaks, including during the COVID-19 pandemic, with healthcare-acquired clusters of severe acute respiratory syndrome coronavirus—2 (SARS-CoV-2) infection reported among patients and staff [1]. HCWs’ compliance with infection prevention and control (IPC) precautions has been consistently reported to be lower across a broad spectrum of precautions [2,3]. A major cause of transmission is poor compliance with personal protective equipment (PPE). The reasons for poor compliance include: working outside of emergency or intensive care settings; not working with confirmed infection cases; lack of concern about risk of infection; lack of monitoring by superiors; observed noncompliance of colleagues; lack of PPE; perceived difficulty using PPE; perceived lack of effectiveness or lack of importance of PPE; perceived inconvenience and discomfort of PPE; perceived negative impact of PPE on patient care; lack of infection control guidance; and inconsistent or unclear guidance [4].

An international survey of HCWs in 2020, found a strong association between reporting never having received PPE training and low confidence in using PPE during the COVID-19 pandemic [5]. Inadequate IPC training and the need to focus on enhancing opportunities for education and training appears to be a recurring theme amongst the studies that have measured the levels of knowledge and preparedness regarding COVID-19 infection control among HCWs in low-resource settings. For example, a study performed in Pakistan recommended that the Health Ministry should provide a comprehensive IPC training program, targeting all HCWs, to promote all precautionary and preventive measures of COVID-19 [6]. Another study, conducted in Libya, found that less than 7% of HCWs received training on how to manage COVID-19, resulting in low infection prevention and control awareness among HCWs [7]. Even in high-resource settings, issues around training have been identified. Between June and September 2020, a cross-sectional study of Australian infection control professionals (ICPs) and infectious disease (ID) physicians, including members of the Australasian College for Infection Prevention and Control (ACIPC) and Australasian Society for Infectious Diseases (ASID), was conducted [8]. The authors found that the respondents identified that keeping up to date with guidelines regarding ‘Contact tracing and outbreak management’, ‘isolation practices’, ‘use of PPE’, and ‘public health orders’ were ‘very difficult’. In addition, not all ICPs (25/100) and ID physicians (*n* = 15/45) reported they had received some form of specific education, training, or instruction about COVID-19 within their workplace. Around 30% reported not feeling ‘highly confident’ about using PPE.

The inclusion of training programs on IPC, in addition to other employee occupational health strategies, can help decrease the risk of infections amongst HCWs [9,10,11,12]. However, a lack of awareness and understanding amongst HCWs regarding the key principles of occupational IPC (OIPC) has been repeatedly demonstrated, even in situations where education and training sessions have been provided [13,14]. In this situation, the focus is often placed on breakdowns within the clinical environment. A 2022 scoping review found similarities, variations, and omissions in the way that OIPC training programs for HCWs are framed across IPC guidelines of low-, middle-, and high-income countries [15]. While studies have reviewed the quality of OIPC training in the healthcare settings, less focus has been given to understanding the factors that impact training delivery. This indicates a need for further investigation to ensure that the IPC training needs of HCWs are being met to protect them from infections at work.

The overall aim of this paper was to examine the current landscape around IPC training of HCWs in the healthcare settings of selected low-, middle-, and high-income countries conducting semi-structured interviews with critical infectious disease policy.

## 2. Materials and Methods

A qualitative approach was adopted to meet the overall aim. Semi-structured, in-depth phone interviews were undertaken with key stakeholders and informants between July 2019 and January 2021. The focus countries included Pakistan, India, Indonesia, the Philippines, the Kingdom of Saudi Arabia, and Australia. To meet the aim, an interview guide was developed by the authors to cover the following focus areas:

1. Structure and delivery of IPC training programs;
2. Resource allocation;
3. Impact of COVID-19 on IPC training programs.

### 2.1. Design

A descriptive design was used, as this approach is said to be ideal for the exploration of the beliefs of different groups of people [16], such as the healthcare staff involved in IPC policy-making or implementation.

### 2.2. Participants and Recruitment

Purposeful sampling was undertaken, in order to identify and recruit relevant stakeholders and ensure diversity in both setting and context. We defined stakeholders as individuals who could communicate in English and were involved with policy-making or the implementation of control strategies for infection prevention and control. This principally encompassed personnel such as those working at in-country Centres for Disease Control and Prevention or at the Ministry/Departments of Health. We also reached out to key research leaders and individuals in other key agencies, such as WHO and relevant colleges/peak bodies. Each participant was sent an email invitation to participate. Consent to participate in the study was sought verbally and recorded on a digital recorder. This study did not collect any identifiable personal information from the participants.

### 2.3. Data Collection and Analysis

Semi-structured interviews were conducted by MO. Questions were asked in an open-ended manner to allow room for expansion. Prompts were only given when the interviewer deemed that they were required to encourage the conversation back to the topic. In-house subject matter experts were consulted to check for the validation of the interview questionnaire. Interviews were audio-recorded, transcribed verbatim, and checked for accuracy by one of the researchers. Interview transcripts were thematically analysed using framework analysis, a method of coding and analysing interview transcripts, designed by NatCen [17]. Framework analysis consists of the following stages: (1) familiarization of the data; (2) identification of thematic framework; (3) indexing; (4) charting; and (5) mapping and interpretation. This method of analysis uses a thematic approach. It allows themes to be developed from the narratives of the interviewees, as well as from the research questions initially posed using the interview guide [18]. It, thus, provides flexibility, while also being systematic. It facilitates the development of a framework for discussing the findings around the themes identified during the analysis.

QSR International’s NVivo 12 qualitative data analysis software was used to code all transcripts, categorise the data, and facilitate a comparison of participant views. A code list of major themes was independently constructed by MOQ and cross-checked by HS and AC. After initial analysis, transcripts and emergent themes/subthemes were iteratively reviewed and modified after further discussion. We identified emerging themes through the analysis of the first 10 interviews, then continued to sample, following identified leads, until we reached thematic saturation. This meant we reached a point where no additional issues or insights emerged from the data, and this redundancy signaled that data collection may cease.

### 2.4. Ethical Considerations

Ethics approval was sought and received from the Human Research Ethics Committee, UNSW Sydney (HC reference number: HC190341). All participants were made aware that the choice to participate or withdraw from the study would not affect their relationship with UNSW Sydney. This information was provided verbally and in writing on the participant information sheet and consent form.

### 2.5. Rigour

To ensure the study’s rigour, HS and AA analyzed a sample of the transcripts. This process enhances reliability and helps set aside any preconceptions by the interviewer [19]. Furthermore, member checking was conducted to enhance the study’s interpretative rigour during the interviews and ensure that the ideas identified during the early analysis phase were appropriate. Debriefing was performed at the end of each interview. The team discussed the findings weekly, and interview guides were modified and revised as needed.

## 3. Results

Twenty-two stakeholders participated in the study (Table 1), including five from the Kingdom of Saudi Arabia, four from Pakistan, four from India, four from Indonesia, two from the Philippines, and three from Australia. One participant from India later withdrew from the study. The themes that were relevant to training are described below.

### 3.1. Training Should Be Mandatory, but There Is Variation in Policy

There was a consensus among the participants that attending IPC training programs should be mandatory for HCWs. However, it was also acknowledged that, currently, attending training is only mandatory for specific groups of staff members, with a focus on those staff who provide direct patient care. Furthermore, it was suggested that only some elements of IPC training were mandatory for HCWs to attend.

“…mandatory for those who are directly involved in infection disease control and management like a doctor, nurse, physician, laboratory worker, even for people in the laundry department [but] those [HCWs] who do not have direct contact it is not mandatory and obligation to attend the training [like] people in the administration section and those who work in non-communicable department …they do not need to attend the training.”

(Participant 22, Indonesia)

“…There would be elements of things that are mandatory, there will be things such as hand hygiene things that everyone is expected to have taken… but again that will vary from hospital to hospital as to what components are mandatory and the frequency of that training being mandatory will be different as well.”

(Participant 19, Australia)

Participants also spoke about the variation between public and private healthcare settings, with one participant acknowledging that attendance is checked in government hospitals, but there is no way to know if the private sector hospitals have mandatory IPC training: “…for the government people [HCWs] yes, it [OPIC training] is mandatory and they are oriented on that but for the private sector there is no check because it is organised by their own people…” (Participant 12, India). Issues were raised about the practical implementation of not only making training mandatory in low-resource settings, but also for tracking or monitoring attendance amongst staff. Concerns were also raised that, if training sessions were mandatory, this could impact patient care in some low-resource settings, due to low doctor-to-patient ratios: “Pakistan is a very large country population wise and it is not easy to cover all these things.” (Participant 14, India).

### 3.2. Preference Is for More Frequent Training, but the Reality Is Different

Aligned to the preference for mandatory training, participants acknowledged that staff members should be undertaking training more frequently. Currently, IPC training programs are usually only delivered once a year. Orientation/induction was indicated as the expected times that training was provided. However, there was undoubtedly variation, regarding the delivery of the training sessions, with some participants acknowledging that training was conducted once every two years (high-resource setting). In contrast, participants from low-resource settings noted that it was not provided regularly.

“…every two years but I think sometimes maybe that doesn’t happen either I know when COVID started we all had to go online and do a PPE course…”

(Participant 15, Australia)

“it is not something which is done on a regular basis”

(Participant 14, Pakistan)

Participants remarked that there was more of a focus on IPC training during an outbreak or pandemic situation, including during COVID-19 or in the past with Middle east respiratory syndrome—coronavirus, in the case of Saudi Arabia.

At the same time, others noted that IPC training was also delivered before the start of the HAJJ season or when the IPC guidelines were updated.

“…healthcare workers are made more aware of putting on PPE if there is actually happening an outbreak or report of a potential outbreak that’s the only time.”

(Participant 11, Philippines)

“…we concentrate on training before HAJJ so all staff will be ready and also all the health workers they are willing to participate in HAJJ. They are not eligible to participate unless they take these [OIPC] courses and we ensure they are taking the courses. So we give like a small card written that he attended, you can look, take care of yourself.”

(Participant 1, Saudi Arabia)

All of the participants agreed that IPC training programs was an important component of preventing HCWs from infectious diseases; however, most pointed out that the attitude of HCWs towards IPC training programs was poor.

“The main problem is not training the main problem probably is making them understand and how important it is for their own health and the health of others, so that is an issue, which needs to be focused on. Behaviour change basically… Generally this is attitude problem through out from top to bottom...the issue is of implementation.”

(Participant 14, Pakistan)

“when you look at how many healthcare workers are currently getting infected with COVID 19, they don’t take it [IPC training] seriously.”

(Participant 16, Australia)

### 3.3. Ability to Deliver Tailored Training Programs

Participants indicated that a range of IPC topics should be included in the training programs, including hand hygiene, adequate donning and doffing of PPE, preventing needle stick injuries, reprocessing of reusable medical equipment, cleaning and disinfection, and waste management. While all of the participants stated that IPC training was delivered on standard and transmission-based precautions, few spoke about the ability to deliver tailored IPC training to the different groups of staff. None of the participants from low-income countries spoke about the delivery of job-specific IPC training for HCWs. In addition, participants acknowledged that there was no specific IPC training for expatriate HCWs when they arrive in a foreign country to work for a healthcare setting.

### 3.4. Classroom Education Approaches Still Dominate

Participants noted that face-to-face, or classroom-based, training was the main method used to deliver IPC training to HCWs, including training staff to don and doff their PPEs. In addition to this, training was also delivered through workshops and seminars. Participants from Saudi Arabia and Pakistan pointed out that, although internet connectivity was no longer an issue, the delivery of training through an online mode was not a common practice, as it is a new concept, and they relied on classroom-based method for the delivery of IPC training. Australian and Indonesian participants pointed out that theoretical part of OIPC training was delivered online, while the practical part of the training program was delivered offline.

“Both ways, online modules that have some basic information, and assessment is part of that and then there is other information that is given in face-to-face training…”

(Participant 15, Australia)

“…healthcare workers sit and listen for most of time, online training and studies is a very new thing. They respond much better in classroom setting.”

(Participant 18, Pakistan)

However, there were mixed feelings regarding the value of online training. Concerns centred around the ability to provide real-time feedback on the HCWs skill set gained were raised.

“[I] do not believe that one-of online training will do the trick in terms of improving practise…there has to be real time feedback”

(Participant 21, Philippines)

### 3.5. Evaluation of OIPC Training Programs

There was an overarching opinion that IPC training program should be evaluated. However, most participants noted that, while IPC training programs are evaluated at the local level, there are no audits conducted on the delivery at the national level.

“This is something lacking their’ s no national oversight of these training programs.”

(Participant 16, Australia)

Furthermore, all participants agreed that feedback from HCWs on improving the quality of the IPC training delivered was important. However, the most prevalent view of all the participants of low-income countries was that feedback from HCWs being trained was neither taken or nor considered for developing and/or updating OIPC training programs.

“No, they [HCWs] are not being asked to participate in the formulation of the [OIPC] training programs. Training programs are being made and being done by the senior officers, who are senior officers across medical colleges and in the ministry also, consultants who are in the ministry. They are doing this making of the training programs. Since they are very much experienced having a lot of knowledge about different aspects and different programs of different diseases. So all these are very high senior officers who are already trained in different aspects and training services.”

(Participant 7, India)

“…the feedback from the participants has never been analysed, they have never tailored [OIPC] training according to the previous feedback… feedback is given [but] they have already designed the training as to what they are going to deliver. The feedback of the healthcare worker is not important so this is the reason no matter how many trainings do we conduct until and unless we listen to their problems and try to cater them they [HCWs] will not listen to us [management]….”

(Participant 18, Pakistan)

The majority of the participants from low-, middle-, and high-income countries acknowledged that HCWs were being assessed for the IPC skills gained after getting IPC trained, but the degree to which they were assessed varied, depending on the type of setting they work in, and this assessment may include evaluation for other aspects of HCWs skills that are also required to deliver healthcare services to patients.

“…sometimes they will evaluate knowledge post-delivery of training. So it will be part of the delivery of training just to quiz people as you go through. so sometimes OIPC training might be competency based, so procedural based there will be that level of checks as well. So it’s going to vary significantly, depending on the type of procedure, the type of setting the people [HCWs] are undertaking”

(Participant 19, Australia)

### 3.6. Lack of Funds to Support IPC Training

Participants emphasized that having a dedicated budget for IPC training programs was important, but almost all participants noted that no dedicated funding was currently allocated. Participants from high-resource settings suggested that the budget for IPC training could be a part of broader package for IPC within the hospital. However, they did note that big public and private hospitals may be able to dedicate resources for IPC training.

“I don’t necessary think that there is budget for IPC training. That budge for [IPC] training would be a part of the package, so for example in IPC service… IPC education and training would be assumed to be part of a wider service which would have a budget line.”

(Participant 19, Australia)

“… it depends for big public hospitals the local government gives them some resources. For private hospitals whether it is a big hospital or only small hospital if it is a big hospital under a big corporation then it has good resources but small hospitals locally owned they do not have enough resources to do that.”

(Participant 20, Indonesia)

Participants from low-resource settings pointed out that IPC training was conducted when foreign funds were made available, as hospitals lacked sufficient budget.

“in routine healthcare care workers do not wear a mask they only wear mask in special settings because also we have limited resources and it is the decision of the hospital how to utilise the available budget for PPE and they did not spend much on PPE… and they are not provided with things as the hospital has limited budget.”

(Participant 18, Pakistan)

“The United states agency for international development funded a lot of training programs on infection prevention and control.”

(Participant 21, Philippines)

### 3.7. Impact of COVID-19 on IPC Training Programs

All of the participants interviewed after the outbreak of COVID-19 agreed that HCWs were unprepared to manage a pandemic such as COVID-19.

“Even if you provide PPE the donning and doffing was done in a wrong way.”

(Participant 17, Pakistan)

“…we developed a pandemic preparedness plan and infection control in hospital setting and those kind of things after the [H1N1] pandemic we did not practise those policies… and when the [COVID-19] pandemic began we were not really prepared…”

(Participant 20, Indonesia)

“A lot of hospitals weren’t prepared. They didn’t have fit testing for example for masks or the process for that. They had to completely restructure and retrain a lot of their staff very quickly.”

(Participant 15, Australia)

The participants noted that, during COVID-19, the perception of HCWs towards protecting themselves improved, and this impacted hand hygiene behaviours and attendance in training.

“… when I was in hospital I never used to sit in the ward or OPD using a mask regularly. It would have been a very rare that I had to use a mask. I think it has bit changed. This COVID-19 has changed the mentality of HCWs.”

(Participant 17, Pakistan)

“A very big change for example in the hospital hand washing compliance was low before COVID-19, the monitoring of health washing compliance significantly increased due to [OIPC].”

(Participant 21, Philippines)

“There is a lot of training going for healthcare workers during COVID-19 pandemic around infection control that’s being driven as part of the risk management process but also healthcare workers really asking for that [OIPC] training. I think that’s been a change I guess perception of increased risk to themselves and quite rightly so in certain areas.”

(Participant 19, Australia)

“…of course because of some [OIPC] training most of them [HCWs] are changing their usual behaviour and try to do better to manage this COVID 19 pandemic.”

(Participant 22, Indonesia)

However, the majority also indicated that more needs to be done, as adequate IPC training is not delivered to HCWs, which could be one reason for the high number of infections among HCWs during COVID-19.

“I don’t think [OIPC training] it is adequate because as you know in Indonesia we have higher infection rate among healthcare workers… That is an example of inadequate training on infection prevention and control for health care workers.”

(Participant 20, Indonesia)

“Clinicians are overburdened, we have huge working hours and do not have continuous medical education type of thing in the hospitals and most of healthcare workers working in hospitals are postgraduate trainees and they are very targeted about completing their milestones.”

(Participant 18, Pakistan)

## 4. Discussion

The participants in our study reported various challenges in training delivery, but all agreed that OIPC training is a critical component of an IPC strategy. The findings outlined in our study resonate with the broader literature regarding the challenges and benefits of IPC training for HCWs [15,20].

In low-resource settings, it has been suggested that the usefulness of OIPC training has historically been limited, due to a range of reasons, including: (1) inadequate and inappropriate utilisation of funding; (2) inability to scale up; (3) poor alignment or tailoring of training resources/approaches to local priorities and settings; (4) insufficient emphasis on the acquisition of practical skills; and (5) lack of coordination [21,22]. A systematic review of studies regarding PPE use for respiratory infections in healthcare settings in Pakistan reported a lack of OIPC training being delivered to most HCWs [23]. Similarly, Saima Hamid et al., in their study conducted in Pakistan to examine and compare job satisfaction among nurses, highlighted that, due to shortage, administration preferred to not send nurses to training courses [24]. The delivery of OIPC training amongst HCWs in high-resource countries does not necessarily predict good OIPC practice [25]. For example, studies conducted before the COVID-19 pandemic reported that, despite the adequate protective controls put in place, including well-established guidelines and training, HCWs have been found to demonstrate poor compliance with hand hygiene practices in countries such as Australia, Canada, France, Finland, Germany, Saudi Arabia, Switzerland, and the United States of America (US) [26,27,28,29,30,31].

While considered a cornerstone of all patient safety and healthcare-associated infection (HAI) prevention and control programs, OIPC training appears to be stuck in the classroom. Amongst our participants, classroom-based training was reported to be the primary mode of delivery of OIPC training to HCWs. Whilst some spoke about the online mode, there was a sense that this was not a popular mode of delivering OIPC training. This finding has been echoed by others, including in a study by Barratt et al., who reported a similar observation in their research of IPC training in Australia and New Zealand [32]. Traditional approaches to teaching IPC concepts (i.e., classroom-based teaching) may fail to stimulate active participation or lead to improvements in the knowledge, attitudes, or skills of the attendees. Further consideration needs to be given to the implementation of learning approaches that recognise the background experience, prior learning, and diversity of HCWs in the development of OIPC training content, style, and mode of delivery. In adopting an educational framework, consideration needs to be given to the structure of the training, regarding whether it is constructively aligned and the training is functional. Innovations in online teaching technology may enhance learning, as well as enable feedback mechanisms to ensure that it is tailoring to the needs of the participants. To support these actions, the training of specialised infection control staff around adult learning principles and the selection of appropriate training modalities, should be considered.

Previous studies have identified significant differences in the OIPC topics covered in the guidelines of low-, middle-, and high-income countries [15]. According to our study, the baseline set of topics recommended for OIPC training includes hand hygiene, adequate donning and doffing of PPE, preventing needle stick injuries, reprocessing of reusable medical equipment, cleaning and disinfection, and waste management. While there remains no consensus on what constitutes the ideal IPC training approach, the need for tailoring and job-specific training is increasingly being recognised. While there remains no consensus amongst the participants in our study, previous attempts have been made to harmonise the IPC courses that are focused on the training of IPC professionals within regions. For example, a European project known as ‘Improving patient safety in Europe’ (IPSE) was launched in 2005 by the Commission’s Directorate–General for Health and Consumers (DGSANCO) [33]. It dealt with many aspects of HAI prevention and control, including training in infection control and HAI epidemiology, with the aim to develop a core curriculum. Five years later, a survey conducted across 33 countries of the EU found that, while increased attention had been paid, there were still critical issues relating to training opportunities, a lack of harmonization, and variations in the available resources and sustainability of IPC programs [34]. Eventually, a set of competencies for IPC and hospital hygiene professionals was released in 2013 [35]. Within the competencies, the need for OIPC training was recognised, with competencies focused on: (1) the evaluation of training needs; (2) the integration of training of new staff; (3) designing training programs for all staff; (4) the need to develop appropriate training processes; and (5) the need for evaluation. Importantly, as part of this work, wiki tools were developed to support the sharing of tools and discussions around IPC training. It is suggested that further investment is still needed, with the possibility of bringing in training organisations to support activities.

The need for tailoring and job-specific training is increasingly being recognised. The need for tailoring was supported by our participants and, in previous studies, focused on the delivery of training in outbreak situations, such as Ebola. Lapses in job-specific OIPC techniques have previously been linked to increased risk for transmission amongst HCWs, including being infected with potentially fatal pathogens, such as Middle East respiratory syndrome coronavirus (MERS-CoV), Ebola virus, and COVID-19 [36,37]. Tailoring accounts for the differences in preparedness at the facility level, variations in HCWs’ roles and their baseline levels of infection control knowledge and training, and differences in the amount and types of infection control supplies (e.g., PPE) that are available to HCWs. To accommodate these issues, the US CDC suggested that settings are assessed for their infection control readiness and training resources be developed that are “action-oriented, modular, accessible on mobile devices for on-demand use, available in multiple formats, and endorsed by key stakeholders” [38].

Feedback from HCWs on the training programs would significantly improve the development and delivery of IPC training. According to Georgia et al., feedback from HCWs is essential to monitor, review, and improve the quality and delivery of training programs [39]. A recent publication also highlighted the importance of regular feedback systems, as well as the need to communicate the reason for the inability to incorporate feedback [40]. Evaluating training programs from a central auditing body and integrating input from HCWs would improve the quality of IPC training programs, leading to the prevention and control of infection and infectious disease amongst HCWs and patients.

Several recommendations for managing the lack of funds for the development and delivery of IPC training programs emerge from this study, including having a dedicated budget or allocation of funding within the IPC budget and making foreign funds available for the delivery of IPC training in low-income countries. This finding resonates with a report published on the training program developed and implemented by Pakistan’s Ministry of National Health Services, Regulations and Coordination (with funding support from the World Health Organization), which also stressed the need for the allocation of appropriate budgets for training in annual development provincial health plans [41].

A strength of this study is that the participants were sampled from six different countries; yet, the overarching themes were consistent across groups. The findings also aligned with previous work conducted, including the findings from Houghton et al. [2] and Hoernke et al. [42], where the inadequate delivery of IPC training to HCWs is concerned. This suggests that challenges to the delivery of adequate IPC training to HCWs may be more widespread than the countries captured in this study and indicates there may be benefits regarding international communities sharing strategies for the delivery of IPC training programs. An obvious limitation of these findings is responder bias. Participants may be the type of people who are already motivated and interested or feel strongly about the topic area, since such individuals would likely be more willing to develop policies and implement adequate IPC training. Our results may underrate the inadequacy of delivering training to HCWs. Additionally, there are probably confounding factors that are unknown and were not considered while conducting the qualitative analysis of the participants’ views. As a qualitative study, it also presents the views, opinions, and perceptions of participants that do not necessarily reflect the views of those who chose not to participate.

## 5. Conclusions

This study revealed variability in the delivery of OIPC training programs within the six high-, low-, and medium-income countries. The results of this study suggest variations regarding the extent and nature of the training delivered to HCWs, leading to the inadequate delivery of IPC training programs, which, in relation, to the COVID-19 pandemic is problematic. More research is needed to establish the essential elements that could underpin the development of training packages, as well as when they should be administered and how/when they should be evaluated.

## Figures and Tables

**Table 1:** Characteristics of participating stakeholders.

Category	Sub-Category	Frequency	Percent (%)
Gender	Male	15	68
	Female	7	32
Nationality	Kingdom of Saudi Arabia	5	22.8
	India	4	18
	Indonesia	4	18
	Pakistan	4	18
	Australia	3	13.7
	Philippines	2	9
Affiliation	Ministry of Health/Department of Health	15	68
	IPC College, association, or peak body	7	32
